# Rapid transmission of respiratory infections within but not between mountain gorilla groups

**DOI:** 10.1038/s41598-021-98969-8

**Published:** 2021-10-07

**Authors:** Robin E. Morrison, Yvonne Mushimiyimana, Tara S. Stoinski, Winnie Eckardt

**Affiliations:** 1 Dian Fossey Gorilla Fund, Musanze, Rwanda; 2 Centre for Research in Animal Behavior, University of Exeter, Exeter, UK

**Keywords:** Ecology, Zoology, Infectious diseases

## Abstract

Minimizing disease transmission between humans and wild apes and controlling outbreaks in ape populations is vital to both ape conservation and human health, but information on the transmission of real infections in wild populations is rare. We analyzed respiratory outbreaks in a subpopulation of wild mountain gorillas (*Gorilla beringei beringei*) between 2004 and 2020. We investigated transmission within groups during 7 outbreaks using social networks based on contact and proximity, and transmission between groups during 15 outbreaks using inter-group encounters, transfers and home range overlap. Patterns of contact and proximity within groups were highly predictable based on gorillas’ age and sex. Disease transmission within groups was rapid with a median estimated basic reproductive number (R0) of 4.18 (min = 1.74, max = 9.42), and transmission was not predicted by the social network. Between groups, encounters and transfers did not appear to have enabled disease transmission and the overlap of groups’ ranges did not predict concurrent outbreaks. Our findings suggest that gorilla social structure, with many strong connections within groups and weak ties between groups, may enable rapid transmission within a group once an infection is present, but limit the transmission of infections between groups.

## Introduction

Human exploitation of the world’s natural resources has led to increased pressure on remaining habitats and increased contact between human and animal populations^1^. This has had a devastating effect on global biodiversity and also creates opportunities for cross-species disease transmission, posing considerable dangers to human and animal populations alike^2–4^. The One Health initiative has highlighted how deeply intertwined the health of human populations, animal populations and their environments are^5,6^. This is particularly critical in relation to our close evolutionary relatives, the nonhuman great apes, who not only are vulnerable to many human pathogens^7^ but have also been the origin of several novel human pathogens^8–11^. The catastrophic effects of infectious diseases such as Ebola Virus Disease (EVD) and those caused by human respiratory pathogens in wild ape populations have been well documented over the past two decades^7,12–18^. Understanding and limiting the transmission of these infectious diseases within ape populations is therefore of high priority when it comes to both the conservation of these critically endangered species^12,19^ and the health of human populations^20,21^.

Across humans and other animals, the social structure of a population can strongly influence patterns of disease transmission^22,23^. This is because social interactions can provide opportunities for pathogens to transmit, such as the transmission of respiratory pathogens through droplets or aerosols during close proximity interactions. By understanding the modes of disease transmission and quantifying these social interactions through networks we can estimate how likely an individual is to come into contact with a pathogen and to transmit it^24–26^. This can provide valuable knowledge to prepare for, prevent or respond to infectious disease outbreaks, and for reducing the prevalence of disease in a population. In chimpanzees, this type of analysis has highlighted the greater risk faced by highly connected community members^27,28^ and indicated that targeted vaccination of the most connected individuals could prevent outbreaks with fewer vaccinations^29^. Despite strong theoretical support linking social structure and disease transmission^24,25,30^, the modelling of real disease outbreaks transmitting through social interactions in wild populations is rarely possible as it requires the long-term collection of fine-scale social data in advance of a potential outbreak^26^.

The long-term data of the Dian Fossey Gorilla Fund includes fine-scale social data and health data on the daily presence or absence of grossly observable signs commonly associated with respiratory illness, for individually-identified mountain gorillas (*Gorilla beringei beringei*). This provides a rare opportunity for investigating the transmission of disease in an endangered, wild great ape, using the presence of these signs as a proxy for respiratory infection. Mountain gorilla numbers have been recovering due to intensive conservation strategies^31^. However, their small total population of roughly 1000, split between two isolated forest fragments makes them extremely vulnerable to extinction from disease^32^. These populations are likely to be regularly exposed to human pathogens due to the dense human populations that surround their habitats and their close proximity to humans during protection, research and ecotourism activities^33,34^. Respiratory disease was reported as the cause of 24% of mountain gorilla deaths—the second most common cause of death after trauma^35,36^. Epidemiologic analyses conducted during three temporally distinct respiratory outbreaks detected pathogens of human origin in symptomatic individuals^37,38^. These respiratory outbreaks were found to be caused by human respiratory syncytial virus and human metapneumovirus, both of which have also been identified in cases of respiratory disease in other ape species^17,39,40^. These studies^37,38^ provide strong evidence that human respiratory pathogens are causing disease in mountain gorillas and that these pathogens are being repeatedly introduced into the population. SARS-CoV2, which can transmit between humans and other apes, including gorillas, may well exacerbate this problem further^41–43^. The recent recovery of the mountain gorilla population may also expose them to even greater disease risk, as their increasing group density within a limited habitat has led to higher rates of interaction between groups and greater home range overlap^44^.

Mountain gorillas’ exposure to human populations also means they pose a particular risk for transferring zoonotic diseases to humans. For example, gorillas have been implicated in multiple spillover events of Ebola virus into human populations^21^. EVD has extremely high mortality in both humans and gorillas^13,45^ and highlights the importance of controlling disease transmission in wild ape populations both for the conservation of those species and the protection of human populations. One EVD outbreak in gorillas has also demonstrated the high costs that sociality can have in relation to disease, with gorillas living in groups suffering higher mortality (97%) compared to solitary males (77%)^45^. To protect both humans and gorillas we need a better understanding of how social interactions influence disease transmission within gorilla groups and across the broader population.

A subpopulation of Virunga mountain gorillas living in the Volcanoes National Park, Rwanda have been closely monitored by researchers at the Dian Fossey Gorilla Fund since 1967. Here, we use fine-scale data on contact and proximity (5 m) collected in 16 groups over 12 years (2004–2015) to identify which individuals may face the greatest exposure to, and capacity to spread infectious pathogens. We then investigate disease transmission within gorilla groups during 7 respiratory outbreaks that took place during this time, determining whether the occurrence of signs of respiratory infection in group members is consistent with disease transmission through the social network. Finally, we use broader-scale data on inter-group encounters, transfers and home range overlap collected between 2004 and 2020 to examine potential pathways of inter-group transmission during 15 respiratory outbreaks.

## Results

### Predicting network position

**Figure 1:**
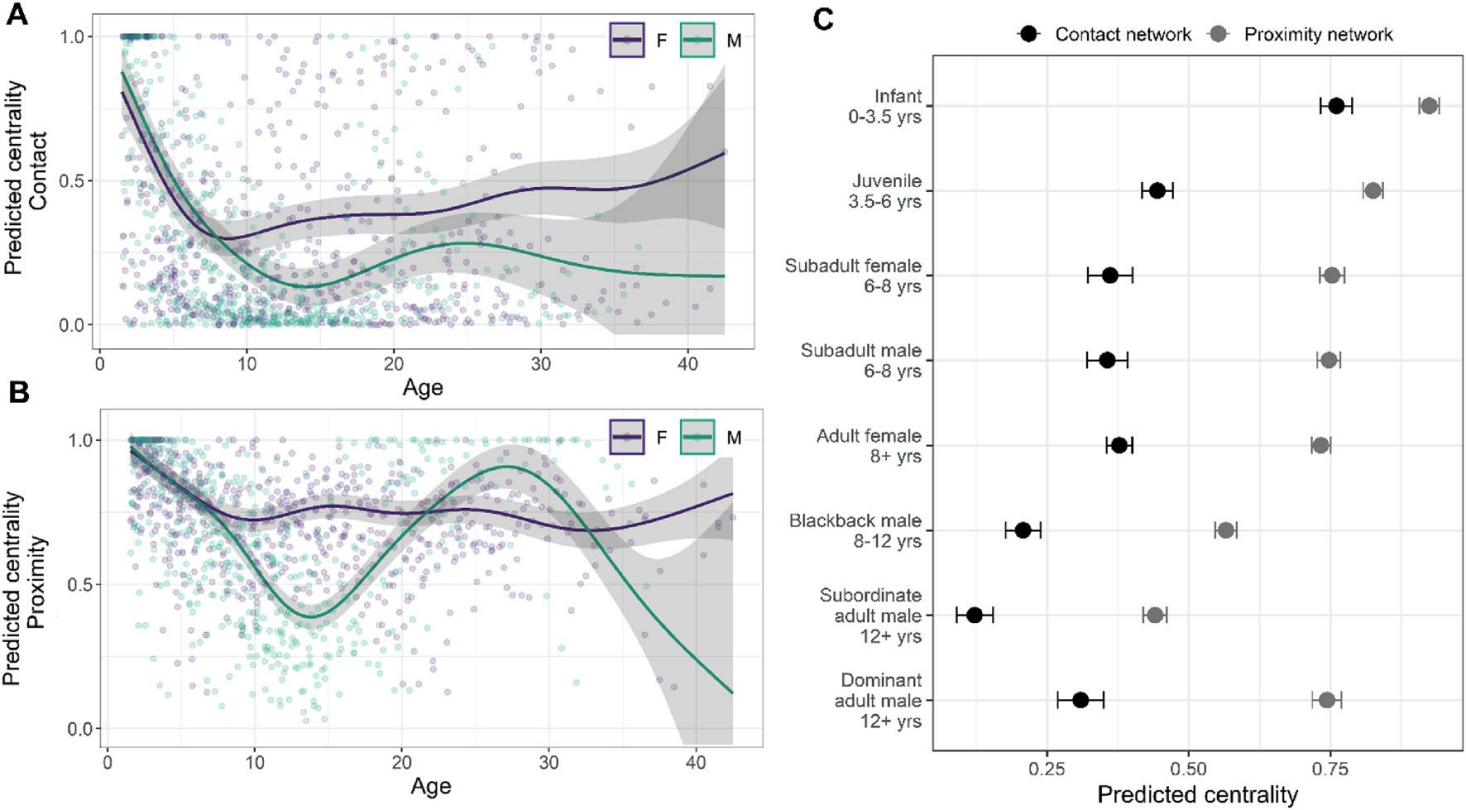
Predicted eigenvector centrality for all individuals in (**A**) contact and (**B**) proximity annual social networks (2004–2015), based on age and sex (F: female or M: male) from GAMMs. Solid lines indicate predicted values, grey shading indicates 95% confidence interval, circles indicate individual data points. (**C**) Predicted eigenvector centrality based on age/sex categories and adult male dominance from linear mixed models in both the contact (black) and proximity (grey) networks. Error bars indicate standard error.

From annual social networks constructed for 12 groups between 2004 and 2015, we extracted individual gorillas’ eigenvector centrality as an estimate of their exposure to, and capacity to spread infectious disease through physical contact and proximity. Using generalized additive mixed models (GAMMs) with smoothed age terms for both sexes, we found that gorillas’ social network positions were strongly predicted by their age and sex (Fig. 1). Linear mixed models using age/sex categories confirmed that infants were more central in both network types than all other age/sex categories (Fig. 1, Supplementary Table S1). Dominance also influenced adult male gorillas’ centrality, with dominant adult males’ having higher centrality than subordinate (t = − 13.951, Pnull < 0.001) and blackback males (t = − 7.005, Pnull < 0.001) in the proximity-based network, and higher centrality than subordinate males (t = − 5.675, Pnull < 0.001) in the contact-based network (Fig. 1, Supplementary Table S1).

### Within-group disease transmission

**Figure 2:**
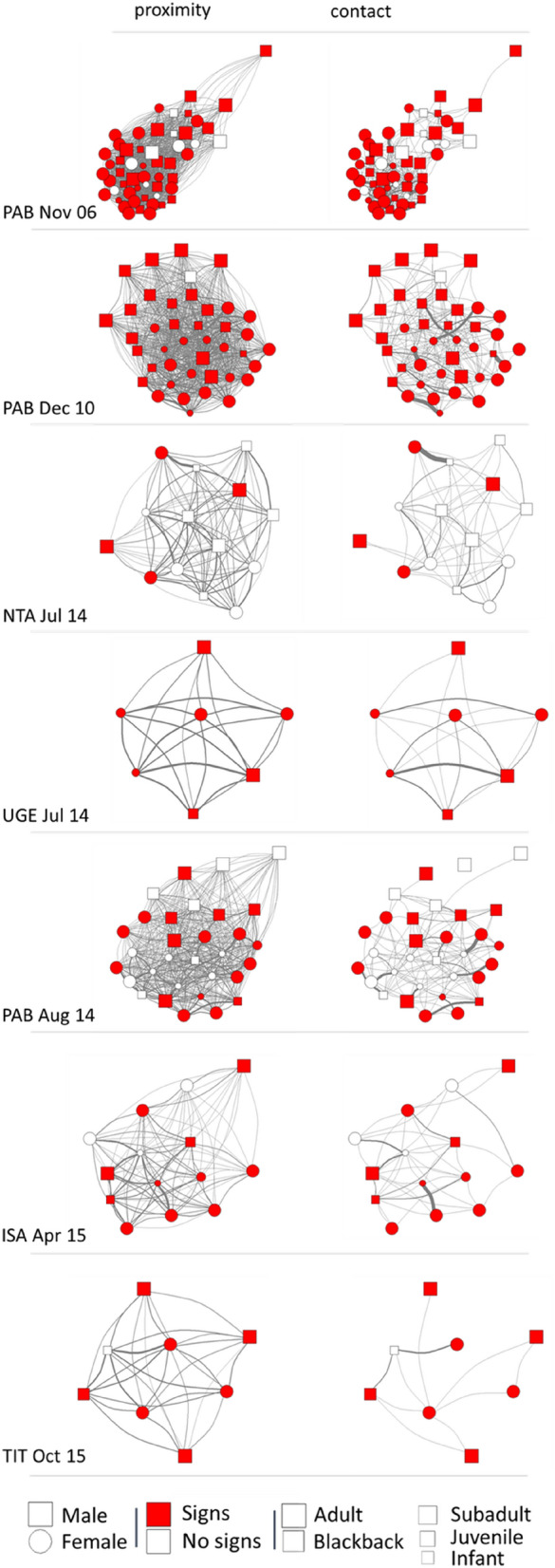
Weighted undirected social networks based on proximity within 5 m and physical contact for each of the 7 outbreaks between 2004 and 2015, based on focal scans collected 3 months either side of the start of the outbreak. Red indicates individuals that were symptomatic at any point during the outbreak. Networks were plotted in igraph using the Fruchterman–Reingold layout algorithm based on the proximity network (to remain consistent between the two network types). The edge width indicates the strength of the dyadic relationship based on the simple ratio index. For visualisations of the temporal spread of signs of respiratory infection in each network, see Supplementary Videos 1–7.

We examined within-group transmission in the 7 respiratory outbreaks that took place between 2004 and 2015 (Table 1, Fig. 2). We built SIR (susceptible-infectious-removed) mathematical models to estimate epidemiologic parameters related to transmission. These suggested rapid transmission of respiratory pathogens was occurring, inferring a median basic reproductive number (R0) of 4.18 (min = 1.74, max = 9.42) across all outbreaks (Table 1).

**Table 1:** Outbreaks (group code, month and year) for which within-group transmission was examined. Transmission period was defined as the number of days from the first group member showing signs of infection to the last day on which a group member showed signs of infection for the first time. Epidemiologic parameters were estimated using SIR mathematical models. R0 refers to the basic reproductive number. Transmission rate refers to the number of subsequent infections per gorilla per day.

Outbreak	Group size	% Infected	Transmission period	Epidemiologic parameter estimates		
				R0	Transmission rate	Infection duration
PAB Nov 06	54	81.5	14 days	3.95	0.94	4.20 days
PAB Dec 10^a^	41	97.6	3 days	–	–	–
NTA Jul 14	14	28.6	6 days	1.74	1.26	1.38 days
UGE Jul 14	7	100	3 days	9.42	2.01	4.69 days
PAB Aug 14	31	58.1	15 days	2.32	0.62	3.77 days
ISA Apr 15	14	78.6	57 days	7.54	0.18	40.85 days
TIT Oct 15	8	87.5	11 days	4.40	0.46	9.64 days

^a^Epidemiologic parameters not estimated due to insufficient data collection to estimate infection duration.

**Figure 3:**
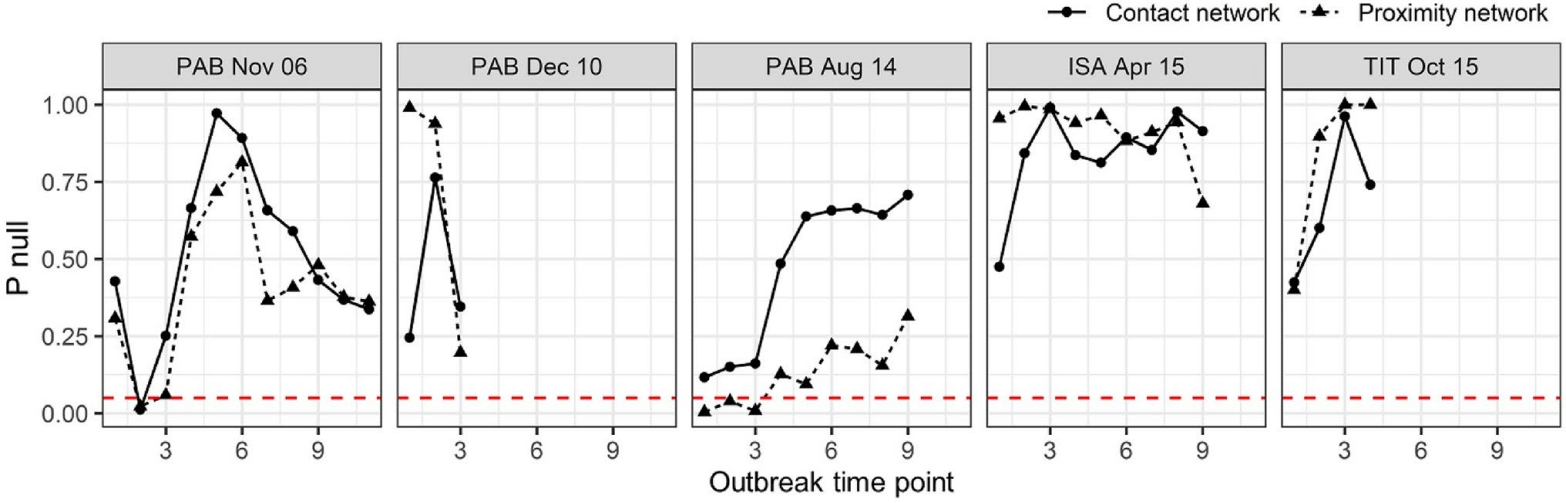
The proportion of null models (P null) for which the mean weighted path length between individuals showing signs of infection was shorter than that observed in the real outbreak, at each outbreak time point (each day of the outbreak on which at least one group member showed signs for the first time) for each outbreak. Outbreaks with fewer than 2 time points were not plotted. Dashed red line indicates a P null of 0.05, i.e. that path lengths between individuals showing signs of infection in the real outbreak were shorter than expected by chance, supportive of transmission through the social network. For visualisations of the spread of signs across each network see the supplementary videos.

We used social networks constructed from contact and proximity data in the 3 months either side of the onset of 7 respiratory outbreaks (Fig. 2) to test whether occurrences of signs of infection were consistent with transmission through the social network. Path-based k-tests were used to calculate the mean path-length between individuals showing signs of respiratory infection in the social network at a given time point. This allowed us to determine whether individuals showing signs of infection were more closely connected to each other than expected by chance—evidence that transmission had occurred through the network. Across the six outbreaks in which not all group members showed signs of infection, those that showed signs were not more closely connected to each other in the social network than expected by chance (Table 2). Those that showed signs were also no more central in their social network than expected by chance (linear mixed models, contact: est = 0.075 ± 0.717, z = 0.104, p = 0.917; proximity: est = − 0.243 ± 0.975, z = − 0.249, p = 0.803). Since it is possible that all group members were ultimately exposed to an infection, the presence or absence of signs of respiratory infection by the end of an outbreak might be determined entirely by an individual’s immunity rather than the transmission pattern itself. We therefore examined the onset of signs more closely by running a path-based k-test at each outbreak time point—each day of the outbreak on which at least one group member showed signs of infection for the first time. If disease was transmitted through the social network but ultimately determined by individual immunity, we might expect a strong signal for transmission through the social network early in the outbreak, decreasing as all individuals were ultimately exposed. This pattern was only observed in the PAB Aug 14 outbreak (minimum p values on Day 1: p = 0.004 for proximity network and p = 0.116 for contact network) and the PAB Nov 06 outbreak (minimum p value on day 2: p = 0.022 for proximity network and p = 0.012 for contact network), out of the five outbreaks in which more than 2 infection time points occurred (Fig. 3). Given the number of tests conducted, the support for transmission of respiratory pathogens through these social networks overall is fairly low.

**Table 2:** Analysis of respiratory disease transmission during outbreaks (group code, month and year) between 2004 and 2015, excluding those where signs of infection were observed in every group member. Mean weighted path length (MWPL) calculated between all group members to show signs of infection during an outbreak. P null is the proportion of null models for which the mean weighted path length between individuals showing signs of infection was shorter than that observed in the real outbreak. For network diagrams of the distribution of signs across each network see Fig. 2.

Outbreak	Proximity network		Contact network	
	MWPL	P null	MWPL	P null
PAB Nov 06	3.690	0.362	24.300	0.337
PAB Dec 10	2.465	0.197	21.290	0.346
NTA Jul 14	3.748	0.963	61.193	0.881
PAB Aug 14	2.984	0.314	31.012	0.708
ISA Apr 15	2.691	0.680	37.547	0.914
TIT Oct 15	2.543	1.000	81.000	0.741

**Figure 4:**
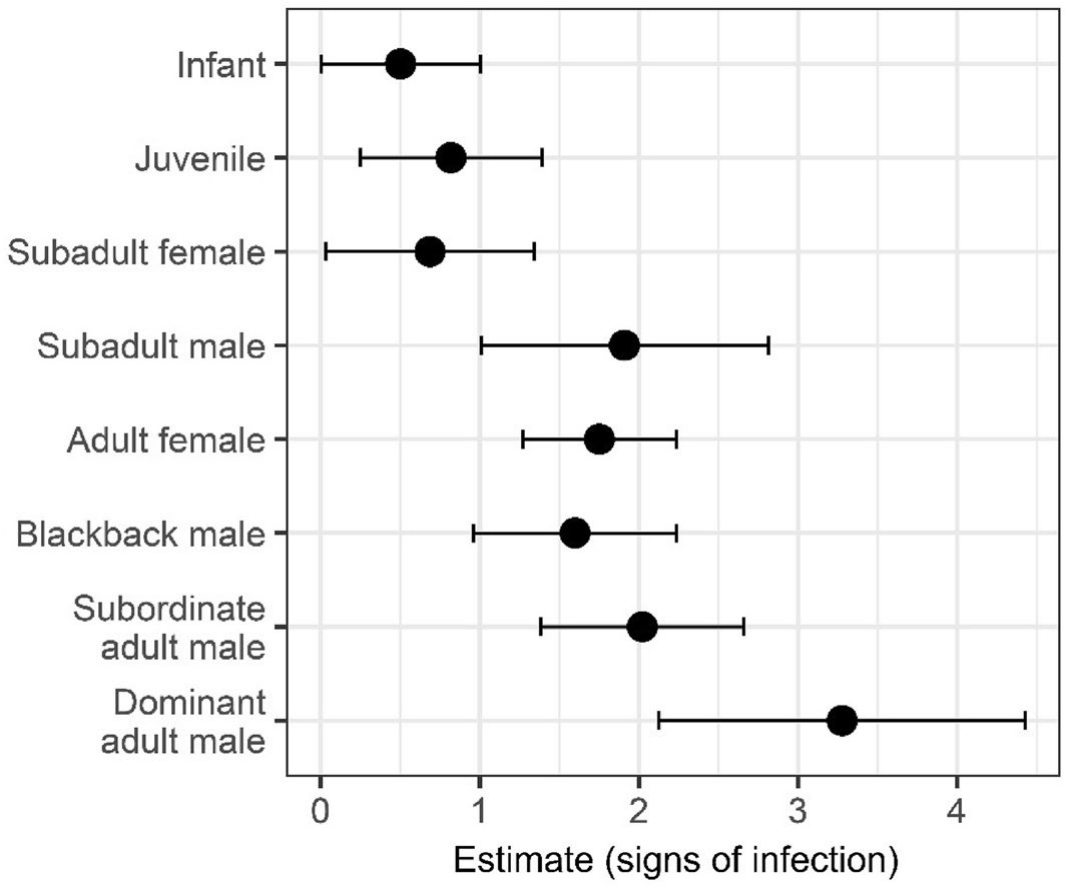
Estimate for the effect of each age/sex category and adult male dominance on whether signs of infection would be observed in a group member during all outbreaks (2004–2020) from binomial linear mixed models. Error bars indicate standard error. N = 274 across 15 respiratory outbreaks.

Examining across all 15 outbreaks (including those after 2015 for which detailed social data were not available), infants, juveniles and subadult females were the least likely to show any signs of respiratory infection (Fig. 4, Supplementary Table S2). Adults were the most likely to show signs, with the highest prevalence seen in dominant adult males. After including an individual’s age/sex category to account for the potential for signs of infection to be more easily observed in some age/sex categories, network centrality for either network type in the smaller subset of outbreaks still did not predict whether a gorilla would show signs of infection during an outbreak (Supplementary Table S3).

### Between-group transmission

**Figure 5:**
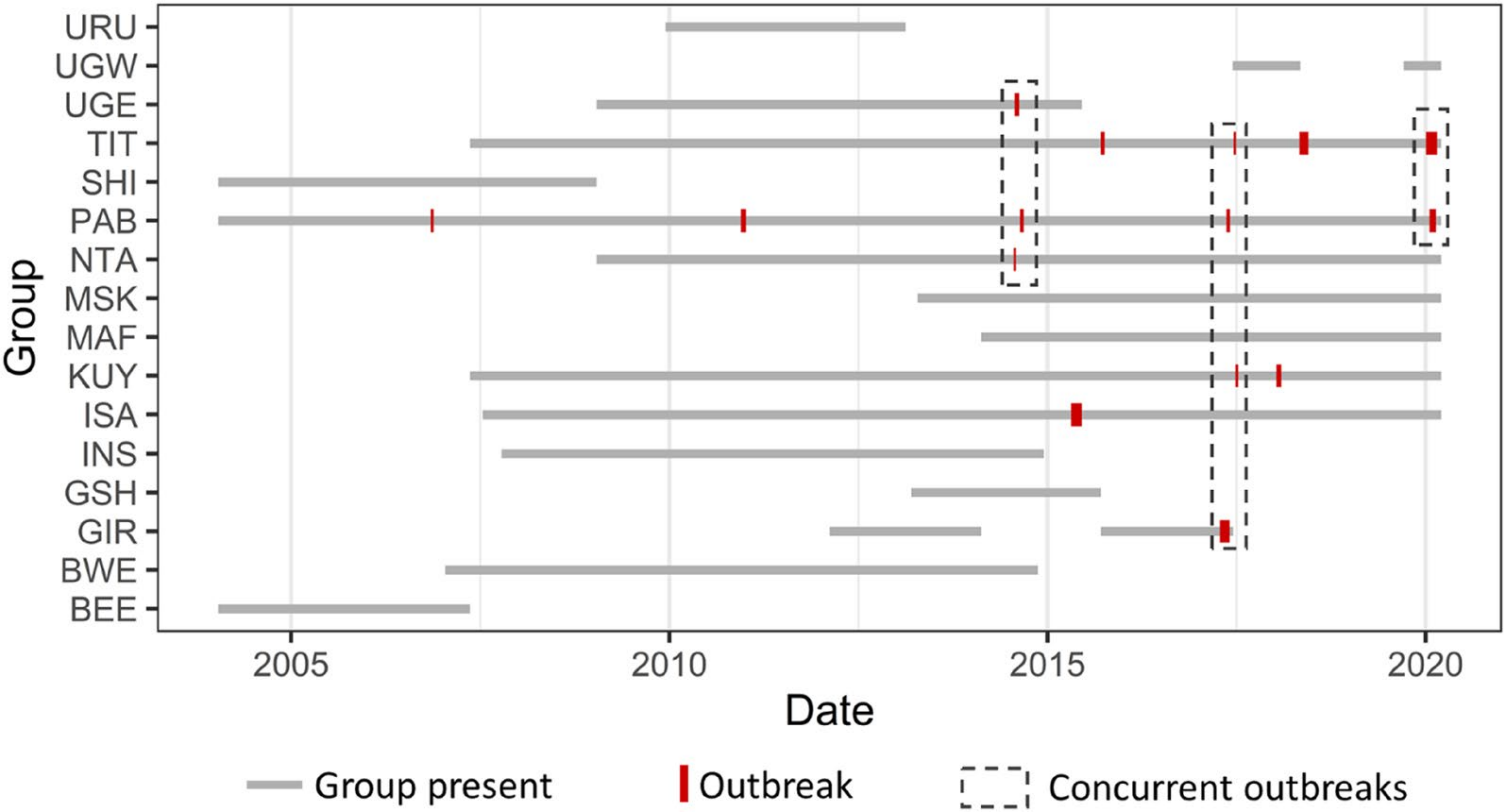
Timing of respiratory disease outbreaks among mountain gorilla groups monitored between 2004 and 2020. Concurrent outbreaks were defined as those that occurred within a month of each other.

Between 2004 and 2020, 15 respiratory outbreaks took place within the monitored groups (Fig. 5). These fell into 9 distinct outbreaks at the population level, based on whether outbreaks occurred in multiple groups within a month of each other (referred to as concurrent outbreaks). Six outbreaks did not occur within a month of those in any other group. Outbreaks occurred once in two groups concurrently, once in three groups concurrently and once in four groups concurrently. This temporal overlap of outbreaks across groups could have occurred through inter-group transmission, through independent introductions from a common source or from independent introductions from multiple sources^17^.

To investigate the potential for inter-group transmission, we examined transfers and encounters between groups. At least one transfer of a gorilla from an infected to an uninfected group took place during 3 out of 15 outbreaks (20%) (Supplementary Information). However, none of these gorillas were showing signs of respiratory infection at the time they transferred into these uninfected groups. Three transfer events took place from a group during an outbreak in which the dominant (and only) adult male died from the infection, resulting in group disintegration. At least one close proximity (< 50 m) encounter with another group occurred during 4 out of 15 group outbreaks (27%). In two of these cases, there was physical contact between members of different groups. In the first case, aggressive physical contact was made between two adult males from the infected group and an adult male of another group. One of these males had not shown any signs of infection during the outbreak and the other had not shown any in 4 days. In the second case, physical contact was made during play behavior between a subadult female from the infected group and a juvenile male from another group. The subadult female had not shown any signs of infection during the outbreak. None of the transfer events or inter-group encounters appear to have led to respiratory disease transmission between groups, as no consistent signs of infection were observed in any individuals in the previously uninfected groups within a month of the event.

As data on inter-group encounters are limited to when observers are present, range overlap during the outbreak period may provide an alternative measure of the probability of two groups encountering one another, and thus, opportunities for inter-group disease transmission. It also provides an estimate for potential environmental transmission, e.g. through feeding at the same location in close succession^46^. In cases of concurrent outbreaks, the ranges of pairs of groups which both had an outbreak did not overlap more than pairs of groups where only one of the pair had an outbreak (binomial generalized linear model: estimate = − 4.954 ± 4.583, z-value = − 1.081, p = 0.280, Supplementary Figure S1, n = 67 pairs of groups). Not only was the result non-significant but the estimated effect was in the opposite direction than expected if inter-group transmission was occurring, with greater range overlap between groups that didn’t have concurrent outbreaks (Supplementary Figure S1). This lack of support for spatial overlap predicting transmission suggests that inter-group transmission of these respiratory pathogens through any of our proposed mechanisms is unlikely to be a cause of these concurrent outbreaks.

## Discussion

We identified strong, predictable variation in gorillas’ network centrality based on their age and sex, and on adult males’ dominance. This means that age, sex and adult male dominance could be used to estimate individuals’ risk of exposure to infectious diseases and their risk of transmitting them. Selective approaches to minimizing disease transmission such as targeted vaccination strategies might therefore provide a valuable conservation tool in some circumstances. However, our analyses of respiratory outbreaks within these gorilla groups found only limited evidence that transmission was occurring through either the proximity or contact social networks (Table 2). Analysis of two outbreaks (out of a total of 5 outbreaks analysed) showed support for transmission through the network early in the outbreak (Fig. 3). But individuals that ultimately became infected during an outbreak were not more closely connected with each other in either type of network than expected by chance and an individual’s centrality in the social network did not predict whether they would become infected. This differs considerably from what has been observed in chimpanzees^27,28^ and many other species^22–24,47–49^ in which social network position can predict infectious disease risk.

This could be for a number of reasons. A key caveat to this study, is that data on the true infection status of individual gorillas were not available. Infection status across this wild and endangered population could only be estimated using the presence or absence of observable signs commonly associated with respiratory illness—an imperfect measure of infection status^50^. Gorillas may vary in the onset of these signs relative to when they are exposed to the infection or in the detectability of these signs. For example, in these outbreaks, signs of infection were more commonly observed in adults, a pattern also observed in chimpanzee respiratory infections^15^. Adults may truly have higher rates of infection or could just be developing more detectable signs. However, even after accounting for age/sex categories and the potentially different detectability of signs within them, network centrality did not predict the presence of signs of respiratory infection. Variation in the immunity of group members could also play a part in obscuring any relationship between sociality and transmission^51,52^. If all group members are rapidly exposed to the infection, the presence or absence of signs may have more to do with their susceptibility than their exposure.

The density of connections within gorilla groups (Fig. 2, Supplementary Videos) also hints at another reason why we may not be able to clearly predict disease transmission through these networks. When networks are so highly interconnected, fine-scale variation in rates of proximity and physical contact could have little real-world consequence, with all group members likely to be rapidly exposed^53^. These networks are also only based on interactions during the daytime and cannot account for potential transmission pathways during the night when group members nest in close proximity^54^. Finally, the relatively small group sizes of mountain gorillas may be limiting the power of such analyses compared to species living in larger social units such as chimpanzees^28^. Both outbreaks where transmission initially appeared to follow the social network were in groups of > 30 gorillas (54 and 31 group members), but no signal for transmission through the network was detected in groups formed of 8, 14 or 41 gorillas. Whilst there is no strong support for transmission following within-group social networks, this is by no means evidence against sociality influencing respiratory disease transmission in gorillas more broadly. At a population scale, sociality clearly remains an important predictor. Most outbreaks occurred in only one group at a time (Fig. 5), with individuals in an infected group at far greater risk of becoming infected than individuals in neighbouring social groups.

Within-group transmission for the majority of outbreaks was extremely rapid. For example, in one outbreak, all 7 members of a group were showing signs of respiratory infection within 3 days (UGE Jul 14). In another, it took only 3 days for 45 out of 46 group members to show signs of infection (PAB Dec 10). Estimated basic reproductive numbers for outbreaks ranged between 1.74 and 9.42. For comparison, an outbreak of human metapneumovirus—one of the pathogens previously found to be responsible for respiratory outbreaks in the population, had a R0 of 1.27 in the Ngogo chimpanzee population^17^. Our findings suggest that the dense connections within gorilla groups may enable respiratory infections to spread so rapidly that we cannot effectively predict or mitigate their transmission within groups.

The highly modular social structure of gorilla populations, with many interactions within groups and few interactions between groups, may provide protection against wider disease transmission, as we found no evidence in support of transmission through the wider population. Transfers and inter-group encounters occurred during 20% and 27% of respiratory outbreaks respectively, suggesting that they could represent important pathways of inter-group transmission. However, neither mechanism appear to have caused any of the 15 outbreaks examined here, as signs of infection were not observed in previously uninfected groups following individuals transferring from, or encounters with an infected group. Where physical contact between members of different groups or transfers occurred during an outbreak, none of the individuals involved were displaying signs of infection at the time, suggesting that this pathway for inter-group transmission is rare, occurring in fewer than 1 in 15 outbreaks (< 6.6%). The ranges of infected groups during concurrent infections also did not overlap more with each other than with non-infected groups. This inability to predict the presence of signs of infection in a group from spatial overlap further supports the lack of inter-group transmission suggested by analysis of direct interactions (encounters and transfers). The minimal opportunities for transmission at this broader scale may prevent most respiratory infections from circulating more widely in the population, limiting their potential conservation impact. However, respiratory outbreaks remain common nonetheless and represent the second most likely cause of death^35,36^. Since our findings suggest inter-group transmission is an unlikely source of these outbreaks, this implies that an alternative pathway is responsible for spreading infections to new groups.

Given the confirmed human origin of a number of respiratory infections in gorillas^37,38^, humans represent the most plausible source of these outbreaks. The frequency of outbreaks and the lack of evidence for inter-group transmission suggests human-gorilla disease transmission may be a regular occurrence within this population. Repeated introduction of respiratory infections from humans, rather than through inter-group transmission has also been found in chimpanzees, where concurrent outbreaks in nearby communities were caused by two distinct respiratory viruses of human origin^17^. These outbreaks can result in considerable mortality, with a human respiratory pathogen outbreak resulting in 8.9% mortality in one chimpanzee population, a far higher rate than that caused by the same pathogen in humans^18^. This highlights the considerable risk from exposure to human diseases that habituated ape populations face, as well as the risk they pose as a potential source of zoonotic disease transmission to humans.

Tourism, research, daily protection and monitoring have been crucial to the intensive conservation strategy responsible for the recovery of the mountain gorilla population^31^ and are used in the conservation of numerous great ape populations. However, these methods also pose some risks of disease transmission between humans and nonhuman great apes^33,55^. Continuing the ongoing efforts to protect wild great apes through extensive testing, vaccination and mask-wearing for all those that come into close proximity with them will be vital, and priority vaccination of human populations close to great ape habitats could further reduce these risks^56^. Given the risk now posed to great apes by the coronavirus pandemic^41–43^, it is more vital than ever to minimize pathways of human-ape disease transmission, which pose a risk to wild great apes and humans alike.

In addition to the respiratory outbreaks examined here, there are a number of other diseases that could pose a threat to gorilla conservation in the coming years including COVID-19 and ebolavirus disease (EVD). Our analyses provide information on disease transmission within wild gorillas which could help in preparation for and in response to outbreaks of such diseases. However, it is important to consider that the mode of transmission and the species between which transmission can occur may differ greatly between pathogens. Given these limitations, our findings can provide only rough guidance for conservation approaches for limiting disease transmission in gorillas more broadly. The predictable variation in contact and proximity based on age, sex and dominance could enable strategies such as targeted vaccination to be effective for pathogens with low transmissibility. But the density of connections within gorilla groups suggests that with most infectious pathogens, all group members are likely to be exposed in a relatively short period of time. Opportunities for disease transmission between groups through transfers, encounters or range overlap appear to be considerably rarer—a pattern also seen in chimpanzees^17^. However, contact with carcasses during outbreaks of high mortality^57^ or contact with other infected species might still enable wider transmission of certain diseases through a population. Measures to prevent or control disease in great apes may therefore be best targeted at preventing disease reaching a group where possible e.g. by preventing initial transmission from humans in the case of anthroponotic disease. This may be considerably more effective than attempting to limit transmission within a group once it is there, where transmission is rapid and unpredictable, or preventing transmission between groups, which is likely to be rare if close inter-group contact is required for transmission.

## Methods

### Ethics statement

This study was non-invasive and strictly observational. All data collection followed the ethical guidelines for gorilla research put in place by the Dian Fossey Gorilla Fund and the Rwanda Development Board.

### Study population

Between 2004 and 2020, 16 mountain gorilla groups in the Volcanoes National Park, Rwanda were monitored daily by trained field assistants and researchers at the Dian Fossey Gorilla Fund. These groups were also regularly visited by tourists and park rangers. Groups were formed of one dominant adult male, multiple adult females and their offspring, and often also contained additional subordinate adult males. Researchers visited each group for up to 4 h per day and individually identified group members by physical characteristics. Long-term monitoring has enabled demographic data, such as dates of birth, sex and maternal kinship to be recorded. All gorillas could therefore be classified into the following age/sex categories: infants 0–3.5 years, juveniles 3.5–6 years, subadult males or females from 6 to 8 years of age, adult females from 8 years, blackback males from 8 to 12 years and adult males from 12 years. Adult males were further categorized into either subordinate adult males or dominant adult males based on their dominance hierarchy. Male dominance was calculated using the Elo-rating method^58,59^ based on observed displacement and avoidance events using the R package ‘EloRating’, version 0.43^60^ as described by Wright et al.^61^.

### Within-group social data

Between 2004 and 2015 data on the social relationships within habituated gorilla groups were recorded through focal sampling. During daily monitoring, researchers chose a focal individual for a 50-min follow by systematically working their way through a randomly ordered list of all group members above the age of one year. If an individual could not be observed (e.g. obscured by dense vegetation), the researcher moved on to the next individual on the list and returned to them the subsequent day. During these focal follows, a scan was completed every 10 min recording all gorillas in physical contact with the focal individual and within 5 m of the focal individual.

### Predicting network position

We constructed weighted, undirected social networks based on proximity within 5 m and physical contact for each group in each year using the focal sampling data. The simple ratio index was used to estimate network edges (pairwise relationships between gorillas), such that edges represented the proportion of scans of either individual in which the pair were observed associating (either in physical contact or within 5 m). Networks included only individuals that were present in the group for at least 11 months of the year, were > 1 year old at the start of the year, and for whom more than 24 focal scans had been recorded that year. We extracted eigenvector centrality (hereafter, centrality) for individuals in both the proximity and contact networks using the ‘igraph’ package in R^62^.

We used generalized additive mixed models (GAMMs) in the ‘gamm4’ R package^63^ to model how centrality in both network types changed with age for both sexes. The response variable was the centrality of each individual per group per year (combining measures across all annual networks). A smoothing term for age by each sex was included as a predictor as well as a smoothing term for the number of focal scans per individual per year to account for any potential differences in centrality driven by sampling effort. Smoothing parameters were fit using REML (restricted maximum likelihood) to avoid overfitting and checked using the gam.check function in the ‘mgcv’ R package^64^. The network (for each group in each year) and the individual identity were included as random effects to account for the non-independence of centrality metrics within the same network and for the same individual in different networks. Age was taken as the individuals’ age at the midpoint of the year.

$$\begin{gathered} Annual\,centrality\,model\,1{:} \hfill \\ gamm4\left( {Centrality \, \sim \, s\left( {Age, \, by \, = \, Sex} \right) \, + \, Sex \, + \, s\left( {Focal \, scans} \right), \, random \, = \, \sim \left( {1|Network} \right) \, + \, \left( {1|Individual} \right)} \right). \hfill \\ \end{gathered}$$

We ran linear mixed models in the ‘lme4’ R package^65^ to assess whether centrality (and the disease risk with which it is linked^24^) could be predicted by age/sex categories (described above). The response variable was the centrality of each individual in each group in each year. Predictors included the age/sex category and both a linear and quadratic term for the number of focal scans to account for any potential differences in centrality driven by sampling effort. The network and the individual were again included as random effects. Age/sex categories were determined based on the individuals’ age at the midpoint of the year.

$$\begin{gathered} Annual\, \, centrality\, \, model \, 2{:} \hfill \\ lmer\left( {Centrality \, \sim \, age/sex \, \,category \, + \, poly\left( {Focal \, \,scans,2} \right) \, + \, \left( {1|Network} \right) \, + \, \left( {1|Individual} \right)} \right). \hfill \\ \end{gathered}$$

As network centrality measures are non-independent, we calculated p-values using node-based permutations. The age/sex category of individuals was permuted within each network to create 10,000 sets of permutations in which individuals’ age/sex categories had been randomized. The same model was then run on these permuted data sets. P-values represented the proportion of these null models with an effect size greater than or equal to that observed in the model run on the real data. Models were run firstly with infants as the reference age/sex category, identifying whether infants had significantly higher centrality than other age/sex categories; and then with dominant males as the reference age/sex category to verify whether dominant adult males had higher centrality than subordinate adult males.

### Respiratory outbreaks

Between January 2004 and August 2020, the presence of any signs of respiratory disease in the monitored gorillas, including coughing, sneezing and nose discharge were recorded each day. From these data, we extracted all cases of respiratory outbreaks within a gorilla group. These were defined as time periods during which at least 25% of group members showed signs of infection and no more than 2 days passed without at least one group member showing signs. For groups of fewer than 12, at least 3 group members had to show signs of respiratory infection to be classified as an outbreak, to avoid chronic cases in a small number of group members being incorrectly classified as a respiratory outbreak. The start of the outbreak was classified as the first day a gorilla showed signs of respiratory infection. The end of the outbreak was classified as the last day on which signs were observed, with no signs in any individuals for at least 3 days subsequently. In total, 15 outbreaks were identified across the 16 groups monitored between 2004 and 2020 (Fig. 5).

### Respiratory infection transmission within groups

Fine-scale data on social contact and proximity within groups were available for the 7 outbreaks that took place between 2004 and 2015. We constructed susceptible-infectious-removed (SIR) models following Scully et al.^18^ for each group in each outbreak using the following set of differential equations:

$$({\text{a}})\quad \frac{dS}{{dt}} = \frac{{ - \beta {\text{SI }}}}{N}\quad ({\text{b}})\quad \frac{dI}{{dt}} = \frac{{ - \beta {\text{SI }}}}{N} - \gamma I\quad ({\text{c}})\quad \frac{dR}{{dt}} = \gamma I,$$

where S is the number of susceptible gorillas, I is the number of infectious gorillas, R is the number of recovered gorillas, β is the daily transmission probability, γ is the recovery rate, and N is the number of gorillas in the group. β and γ were estimated by fitting SIR models to the observed incidence data for the presence of signs of respiratory infection during each outbreak. The basic reproductive number, R0, was calculated as *R*0 = $\frac{\upbeta }{\upgamma }$. For graphs of SIR model predictions plotted over data from observations of infection signs see Supplementary Figure S2.

Social networks based on 5 m proximity and physical contact were constructed for each outbreak using focal scans collected in the 3 months either side of the date on which the outbreak started (6 months total). The simple ratio index was again used to estimate network edges. Networks included only individuals that were present during the outbreak, were > 1 year old during the outbreak, and for whom more than 24 focal scans were available during the 6-month period. We ran path-based k-tests^50^ using the test.path function from the ‘k-test’ github repository^66^ with 10,000 iterations. These analyses assessed whether the mean weighted path length (MWPL) between individuals showing signs of respiratory infection was smaller than expected by chance, and therefore the extent to which the social network predicted the occurrence of these signs. These were run on both the proximity and contact-based networks for each group outbreak. These models were first used to determine whether individuals that had shown any signs during the outbreak were more closely connected than expected by chance. This was not done for the UGE Jul 14 outbreak since all group members had shown signs by the end of the outbreak. As all group members may ultimately be exposed to an infection due to the dense connections within group networks, we then examined the onset of signs of respiratory infection in more detail. This was done by running path-based k-tests for each day of the outbreak on which at least one group member showed signs for the first time, assessing whether the group members that had shown signs so far in the outbreak were more closely connected than expected by chance. This enabled us to detect whether there was any evidence for transmission through the social network at any time point during the outbreak.

We used linear mixed models with a binomial distribution to predict whether an individual would show signs of infection during an outbreak. In the first set of models, we predicted whether signs would be observed based only on an individual’s centrality including the specific outbreak as a random effect. These assessed whether more central individuals were more likely to become infected and used only outbreaks for which social data was available. In the second model, we predicted whether signs of infection would be observed in a gorilla based on their age/sex category, this time expanding the analyses to include all 15 outbreaks (2004–2020), as fine-scale social data was not required. Again, the specific outbreak was included as a random effect. As age/sex category-based differences in the likelihood of observing signs in an infected individual could potentially be obscuring an effect of centrality on the likelihood of becoming infected, we then ran a third model for the 7 outbreaks with social data available, predicting whether signs of respiratory infection would be observed based on both the individual’s network centrality and their age/sex category, again with the outbreak included as a random effect.

### Between-group transmission

Across the entire study period (2004–2020) up to 4 GPS locations (location of nest site, location when researchers found the group, location at noon and location when researchers left the group), any encounters with other groups or solitary males and any transfer of individuals into or out of the group were recorded for each group each day. All transfers that took place from a group during an outbreak were extracted from this dataset. Health records of the group that they transferred into were checked to determine whether the transfer event could have enabled disease transmission between the two groups. Health records of the individual transferring were also checked to determine whether they were showing signs of respiratory infection leading up to and after the transfer. All encounters with another group or solitary at proximities of less than 50 m during an outbreak were also extracted. The health records of the groups involved were then checked to verify whether the presence of any signs of infection in these groups indicated that respiratory disease transmission could have occurred during the encounter.

Cases where outbreaks were detected in more than one group within a month of each other were defined as concurrent cases^35^ (Fig. 5). To investigate whether there was a spatial correlation between the ranges of concurrently infected groups, GPS locations of all groups during concurrent outbreaks were extracted. These were extracted from the start of the first group outbreak (date on which a member of that group first showed signs of infection) until the start of the last group outbreak within the overall set of concurrent outbreaks. This covered the total time period during which groups could potentially have infected each other. When this period was less than one month in total, the period was extended to one month from the start of the first outbreak to enable adequate location data points for range estimation. Home range utilization distributions for all groups during these concurrent outbreak periods were estimated using the biased random bridge approach in the ‘adehabitatHR' R package^67^. This approach explicitly modelled successive relocations following a biased random walk. We restricted this to relocations of within 72 h and of more than 50 m. The smoothing parameter was set to 100 following Caillaud et al.^44^, with the diffusion coefficient estimated using maximum likelihood. The overlap of the home range utilization distributions of each pair of groups was calculated using the utilization distribution overlap index in the ‘adehabitatHR' R package^67^. A binomial generalized linear model was then used to predict whether the ranges of infected groups overlapped more with other infected groups than they did with uninfected groups. This enabled us to determine whether concurrent outbreaks were occurring in groups that had greater home range overlap during the period of the outbreaks, suggestive of inter-group transmission through either direct encounters or spatial overlap.

## Supplementary Information

Supplementary Information 1.

Supplementary Information 2.

Supplementary Information 3.

Supplementary Information 4.

Supplementary Information 5.

Supplementary Information 6.

Supplementary Information 7.

Supplementary Information 8.

Supplementary Information 9.

Supplementary Information 10.

Supplementary Information 11.

Supplementary Video 1.

Supplementary Video 2.

Supplementary Video 3.

Supplementary Video 4.

Supplementary Video 5.

Supplementary Video 6.

Supplementary Video 7.

Supplementary Video 8.

Supplementary Video 9.

Supplementary Video 10.

Supplementary Video 11.

Supplementary Video 12.

Supplementary Video 13.

Supplementary Video 14.
