# Supplementary material for: Rapid transmission of respiratory infections within but not between mountain gorilla groups
Source: Sci Rep. 2021 Oct 7;11:19622. doi: 10.1038/s41598-021-98969-8 (PMC8497490; doi:10.1038/s41598-021-98969-8)
Supplement: Supplementary file 8 — Supplementary Information 8. [file 41598_2021_98969_MOESM8_ESM.docx]

**Supplementary Information**

Transfer events

In the first case, an adult female transferred directly from ISA group (infected) to GSH group (uninfected) towards the end of an outbreak. She had last shown signs of respiratory infection 8 days before the transfer and no members of the new group showed signs within a month of her arrival.

Three further transfers took place from an infected group (GIR) when the dominant male of the group became sick and eventually died. One female showing no signs of respiratory infection transferred directly to another group during an intergroup encounter, prior to the death of the dominant male. No new group members showed any signs within a month of her arrival. After the dominant male left GIR group and subsequently died, two adult females with dependent 1-year-old infants remained. One mother-infant pair transferred into another group 4 days after the death of the dominant male. At that time point, neither individual had shown any signs of respiratory infection in over a month. The dominant male of the new group began showing signs of respiratory infection 2 days after their arrival which continued for roughly 2 weeks, but no signs were observed in any other group members. This male also had a history of chronic coughing suggesting the source of this coughing was not an infection spread by these individuals. The transferring infant died through infanticide within a month of joining the group. The other mother-infant pair remained as an independent unit and did not join another group– extremely unusual behaviour for female gorillas. It is thought this behaviour may have been driven by infanticide-avoidance. The female did not join a male until 7 months later when her infant was no longer observed and assumed dead.

A final transfer took place from another infected group (PAB 17). A mother transferred out of an infected group with her infant four days after she had last shown any signs of respiratory infection. The mother-infant pair was not initially found – they could have been solitary or have transferred temporarily to an unmonitored group. A month later the adult female turned up in an uninfected, monitored group without her infant. No members of her new group showed any signs of respiratory infection within a month of her arrival.

**Table S1.** Predicting centrality by age/sex category and male dominance in the contact and proximity social networks using linear mixed models and permutations, with a) infants as the reference category and b) dominant adult males as the reference category (n=1111 from 166 individual gorillas in 12 groups across 12 years)

| **A) Infant as reference category** | | | | | | | | | | | | | |
| --- | --- | --- | --- | --- | --- | --- | --- | --- | --- | --- | --- | --- | --- |
|  | | **Contact*** | | | | | | **Proximity** | | | | | |
|  | | Est ± SE | | t | | P null | | Est ± SE | | t | | P null | |
| Intercept | | 0.853 ± 0.025 | | 33.464 | |  | | 0.924 ± 0.017 | | 53.067 | |  | |
| Juvenile | | -0.229 ± 0.025 | | -9.277 | | **<0.001** | | -0.098 ± 0.014 | | -7.247 | | **<0.001** | |
| Subadult female | | -0.303 ± 0.036 | | -8.472 | | **<0.001** | | -0.171 ± 0.020 | | -8.490 | | **<0.001** | |
| Subadult male | | -0.305 ± 0.032 | | -9.438 | | **<0.001** | | -0.176 ± 0.018 | | -9.587 | | **<0.001** | |
| Adult female | | -0.315 ± 0.024 | | -13.255 | | **<0.001** | | -0.190 ± 0.017 | | -11.367 | | **<0.001** | |
| Blackback male | | -0.476 ± 0.029 | | -16.406 | | **0.001** | | -0.356 ± 0.018 | | -20.148 | | **0.001** | |
| Subordinate adult male | | -0.586 ± 0.031 | | -18.887 | | **<0.001** | | -0.480 ± 0.021 | | -23.354 | | **<0.001** | |
| Dominant adult male | | -0.385 ± 0.038 | | -9.833 | | **<0.001** | | -0.181 ± 0.027 | | -6.788 | | **<0.001** | |
| Focals | | 0.399 ± 0.338 | | 1.179 | |  | | 1.522 ± 0.212 | | 7.190 | |  | |
| Focals^2^ | | -0.537 ± 0.275 | | -1.954 | |  | | -0.709 ± 0.155 | | -4.565 | |  | |
| **B) Dominant adult males as reference category** | | | | | | | | | | | | |  |
|  | **Contact*** | | | | | | **Proximity** | | | | | |  |
|  | Est ± SE | | t | | P null | | Est ± SE | | t | | P null | |  |
| Intercept | 0.468 ± 0.037 | | 12.692 | |  | | 0.744 ± 0.025 | | 29.224 | |  | |  |
| Infant | 0.385 ± 0.039 | | 9.833 | | **<0.001** | | 0.181 ± 0.027 | | 6.788 | | **<0.001** | |  |
| Juvenile | 0.156 ± 0.039 | | 4.025 | | **<0.001** | | 0.083 ± 0.026 | | 3.179 | | **<0.001** | |  |
| Subadult female | 0.082 ± 0.046 | | 1.782 | | **0.043** | | 0.010 ± 0.030 | | 0.338 | | 0.523 | |  |
| Subadult male | 0.080 ± 0.043 | | 1.861 | | **0.020** | | 0.005 ± 0.027 | | 0.194 | | 0.682 | |  |
| Adult female | 0.070 ± 0.036 | | 1.955 | | **0.020** | | -0.009 ± 0.026 | | -0.354 | | 0.971 | |  |
| Blackback male | -0.091 ± 0.040 | | -2.298 | | 0.062 | | -0.175 ± 0.025 | | -7.005 | | **<0.001** | |  |
| Subordinate adult male | -0.201 ± 0.035 | | -5.675 | | **<0.001** | | -0.298 ± 0.021 | | -13.951 | | **<0.001** | |  |
| Focals | 0.399 ± 0.338 | | 1.179 | |  | | 1.522 ± 0.212 | | 7.190 | |  | |  |
| Focals^2^ | -0.537 ± 0.275 | | -1.954 | |  | | -0.710 ± 0.155 | | -4.565 | |  | |  |

*Centrality in affiliative contact square rooted to improve normality of residual. Not the case for proximity.

**Table S2.** Predicting the observation of signs of respiratory infection in group members (presence/absence) across all outbreaks, based on age/sex category and adult male dominance in the contact and proximity social networks using binomial linear mixed models with infants as the reference category (n=274 across 15 outbreaks).

|  | Est ± SE | Z value | P value |  |
| --- | --- | --- | --- | --- |
| Intercept | 0.503 ± 0.498 | 1.009 | 0.313 |  |
| Juvenile | 0.316 ± 0.549 | 0.577 | 0.564 |  |
| Subadult female | 0.185 ± 0.635 | 0.291 | 0.771 |  |
| Subadult male | 1.407 ± 0.901 | 1.561 | 0.119 |  |
| Adult female | 1.250 ± 0.464 | 2.693 | **0.007** |  |
| Blackback male | 1.096 ± 0.628 | 1.745 | 0.081 |  |
| Subordinate adult male | 1.519 ± 0.640 | 2.376 | **0.018** |  |
| Dominant adult male | 2.776 ± 1.154 | 2.406 | **0.016** |  |

**Table S3.** Predicting the observation of signs of respiratory infection (presence/absence) in group members during an outbreak based on individuals’ centrality and age/sex category in the contact and proximity social networks using binomial linear mixed models with infants as the reference age/sex category. Subordinate and dominant adult males combined in single ‘adult male’ category to enable model convergence (n=169 across 7 outbreaks)

|  | **Contact** | | | **Proximity** | | |
| --- | --- | --- | --- | --- | --- | --- |
|  | Est ± SE | z | P | Est ± SE | z | P |
| Intercept | -0.509 ± 1.097 | -0.464 | 0.643 | -0.360 ± 1.318 | -0.273 | 0.785 |
| Centrality | 1.412 ± 0.989 | 1.428 | 0.153 | 1.122 ± 1.273 | 0.882 | 0.378 |
| Juvenile | 0.523 ± 0.826 | 0.634 | 0.526 | 0.156 ± 0.756 | 0.207 | 0.836 |
| Subadult female | 1.091 ± 1.016 | 1.074 | 0.283 | 0.699 ± 0.950 | 0.736 | 0.462 |
| Subadult male | 2.215 ± 1.463 | 1.514 | 0.130 | 1.969 ± 1.432 | 1.375 | 0.169 |
| Adult female | 2.362 ± 0.862 | 2.741 | **0.006** | 1.974 ± 0.775 | 2.546 | **0.011** |
| Blackback male | 1.624 ± 1.010 | 1.607 | 0.108 | 1.219 ± 0.945 | 1.290 | 0.197 |
| Adult male | 2.280 ± 1.022 | 2.232 | **0.026** | 1.847 ± 0.959 | 1.925 | 0.054 |


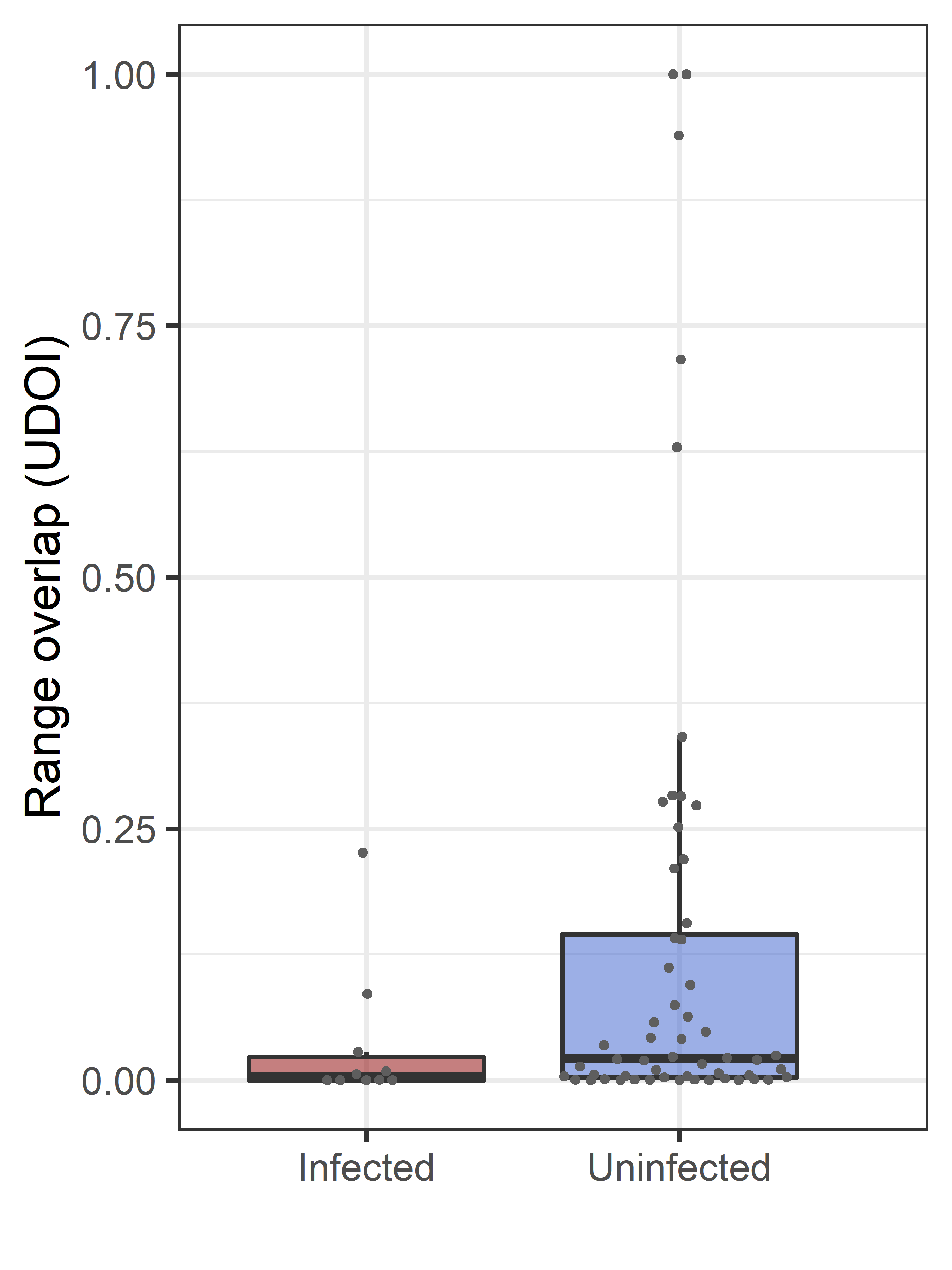


**Figure S1.** Range overlap between pairs of groups estimated by utilization distribution overlap index (UDOI) during concurrent outbreaks where both groups were infected (infected) or where one group became infected and the other did not (uninfected).


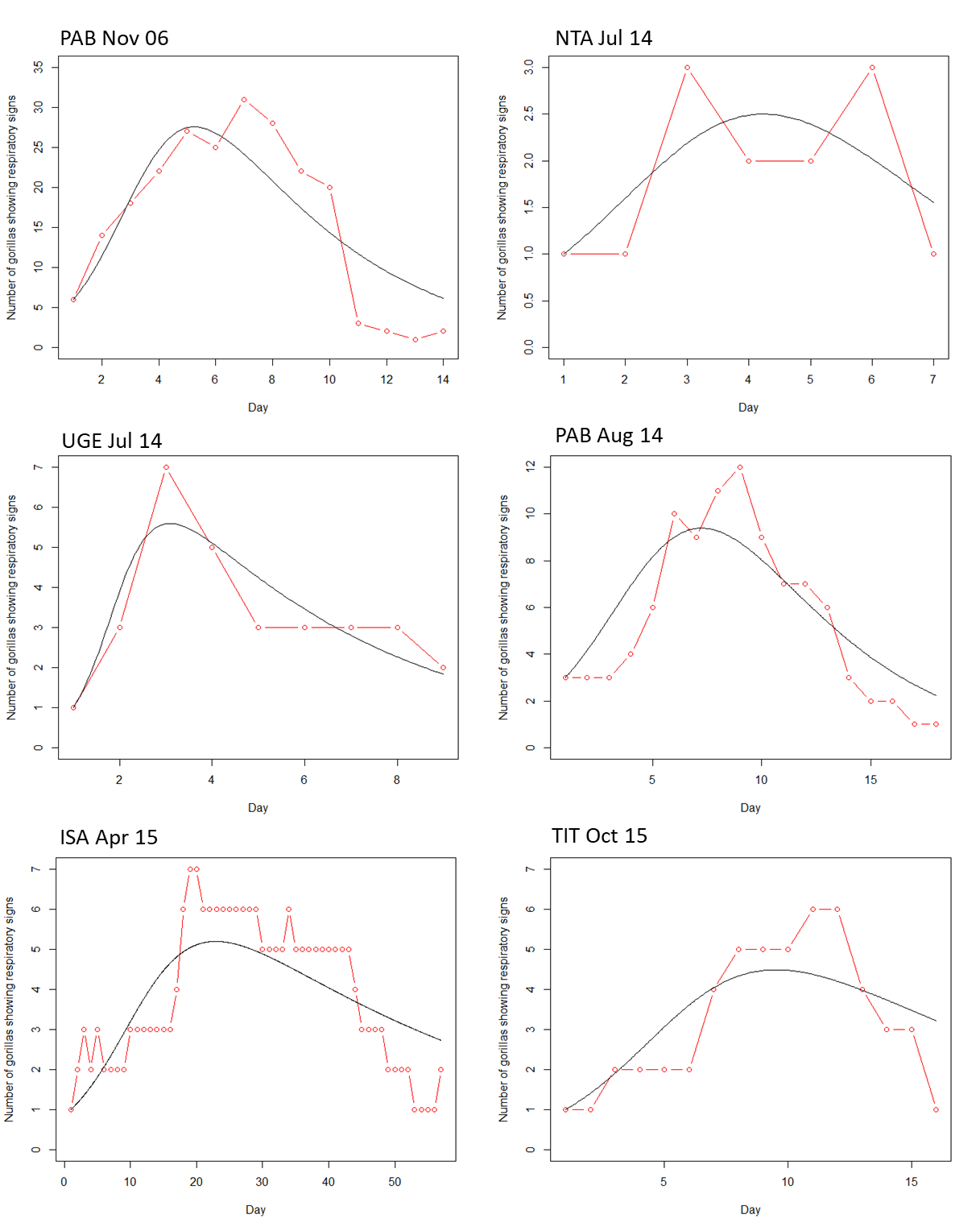


**Figure S2.** SIR model predictions (black) plotted over data from observations of signs of respiratory infection (red) across the outbreak period (day of outbreak) for each of the six outbreaks between 2004 and 2015 for which adequate data was available to build SIR models.

**Supplementary Videos 1-7.** The temporal spread of signs of respiratory infection across a) proximity networks and b) contact networks during each of the seven outbreaks between 2004 and 2015 for which detailed within group social behaviour was available. Red indicates individuals that have shown infection signs within the outbreak period so far. Circles indicate females, squares indicate males. The relative size of these nodes indicates the age of the individual gorilla.
